# Review of Thermophysical Property Data of Octadecane for Phase-Change Studies

**DOI:** 10.3390/ma12182974

**Published:** 2019-09-14

**Authors:** Moritz Faden, Stephan Höhlein, Joschka Wanner, Andreas König-Haagen, Dieter Brüggemann

**Affiliations:** 1 Chair of Engineering Thermodynamics and Transport Processes (LTTT), Center of Energy Technology (ZET), University of Bayreuth, Universitätsstraße 30, 95440 Bayreuth, Germany; Stephan.Hoehlein@uni-bayreuth.de (S.H.); Andreas.Koenig-Haagen@uni-bayreuth.de (A.K.-H.); Brueggemann@uni-bayreuth.de (D.B.); 2 Chair of Empirical Economics, University of Bayreuth, Universitätsstraße 30, 95440 Bayreuth, Germany; Joschka.Wanner@uni-bayreuth.de

**Keywords:** PCM, melting point, latent heat of fusion, density, thermal conductivity, heat capacity, viscosity

## Abstract

In this work we derive temperature-dependent functions for the most important material properties needed for phase change studies with octadecane. Over 80 references are reviewed in which at least one thermophysical property of octadecane is measured. The functions are valid ±40 K around the melting temperature and are surrounded by their confidence interval. It turns out that the values for the solid phase have much broader confidence intervals than the ones of the liquid phase. Hence, more accurate measurements are particularly desirable for the solid state material properties.

## 1. Introduction

The rising CO${}_{2}$ concentration in the atmosphere and the looming climate change have led to a mentality change in power and heat supply, away from fossil fuels to regenerative sources. The most regenerative sources, however, have the disadvantage of fluctuations. Therefore energy storage systems, which are e.g., based on thermal, electrochemical or mechanical effects, are necessary for a reliable energy supply. One important subclass of thermal storage systems are latent heat thermal energy systems, which mostly use the solid-liquid phase transition of a phase change material (PCM) to store thermal energy at a nearly constant temperature. The dimensioning of such systems is mainly done with semi-empirical or numerical methods which need the thermophysical properties of the PCM as an input paramenter. Often these methods are insufficiently validated. This is due to the lack of reliable validation experiments and the strong scatter of available literature values for one and the same thermophysical property.

There are a lot of different PCMs available on the market, but octadecane (C${}_{18}$H${}_{38}$) is one of the most studied PCM and is often used for validation experiments [1,2,3,4]. Frequently mentioned reasons for this are the melting temperature close to the ambient conditions, a translucent liquid phase, cycle stability and apparently well-known thermophysical properties [5]. Nevertheless, the thermophysical properties of octadecane given in the literature vary greatly, especially close to the melting temperature and in the solid phase. The reason for this is that paraffins were first studied in the petrochemical industry [6], i.e., at higher temperatures where the paraffins are liquid and that measuring close to or across a phase transition is far from trivial [7,8]. Near the melting point, the change in the thermophysical properties is very rapid and not all measuring methods can cope with this additional difficulty. A complete literature review including the measuring methods will be given in Section 2.

Obviously, the uncertainty in the input parameters, i.e., the thermophysical properties, propagates through the empirical or numerical model [9] and makes it hard to distinguish between numerical errors, modelling errors and errors due to wrong input parameters. This makes validation more difficult and reduces its usefulness.

In the late 90s Bertrand et al. [10] compared numerical methods for liquid-solid phase change methods and utilised rather crude thermophysical properties for octadecane. Although it is certainly useful to check for numerical errors by applying standardized thermophysical properties, no later than during the validation of the numerical model with experiments the best possible approximation of the real thermophysical properties of the involved PCM are needed [11]. Sometimes missing properties are supplemented with data from similar materials. For example Kozak and Ziskind [12] took the, also uncertain, thermophysical properties of eicosane and applied them to octadecane - although they differ in the single digit percentage range.

Another issue is that the majority of authors neglect the density change during melting and use the Boussinesq approximation to model natural convection, despite the fact that there are methods for solving melting or solidification with volume change [13,14]. In addition, many authors assume temperature-independent properties, although they can vary quite strongly, e.g., the viscosity. Maybe this is because the influence of the thermophysical properties is underestimated. Tan et al. [15] and Madruga and Curbelo [16] investigate the complex spatio-temporal behaviour of solid-liquid phase change coupled with fluid flow, whereas the former assume a constant thermophysical property value regardless of the phase and the latter distinguish between constant values in the solid and liquid phase. A positive exception is Galione et al. [17], who simulate the melting of octadecane with temperature-dependent values for most properties. To do so, they derive linear functions, but the selection of the raw data is arbitrary and only one reference per property is used to derive the function.

The aim of this study is to reduce the uncertainty about the thermophysical properties of octadecane and relief the modeler of a cumbersome literature review. Based on a comprehensive review of data available in the literature and own measurements, we provide estimates for the melting temperature and enthalpy, as well as for the relationships between temperature and thermal conductivity, heat capacity, density and viscosity. These are the most relevant thermophysical properties to simulate melting and/or solidification processes. Another important parameter is the volumetric coefficient of thermal expansion, which can be derived from the determined density curves and is therefore not considered separately. In addition, uncertainty bounds for each property are specified by means of statistical methods applied to the raw data of the literature.

## 2. Literature Review of Thermophysical Properties of Octadecane

This section provides an overview of the available literature data on the thermophysical properties of octadecane. If not available, the uncertainties required for the statistical evaluation are estimated from the applied measurement methods.

### 2.1. Melting Temperature

**Figure 1:**
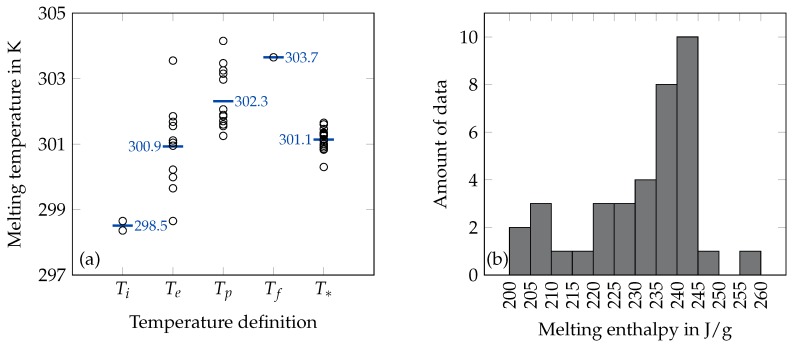
Summary of melting temperature (**a**) and enthalpy (**b**) data from the literature. The melting temperature (**a**) is arranged according to the given temperature definition. The blue bars represent the mean value of the data at each temperature definition. The melting enthalpy data (**b**) are presented in the histogram according to their frequency.

There are numerous results for the melting temperature of octadecane available in the literature. A lot of these results were achieved from the heat flow signal of differential scanning calorimetry (DSC) measurements but without giving information about the way of determining this temperature. Höhne et al. [18] describe five characteristic temperatures of the heat flow signal of a melting process which are the initial peak temperature $T_{i}$, the extrapolated onset temperature $T_{e}$, the peak maximum temperature $T_{p}$, the extrapolated offset temperature $T_{c}$ and the final peak temperature $T_{f}$. The difference between these temperatures depends on the sample and test parameters (thermal conductivity, mass, heating rate). This can be one reason for the huge spread of several kelvin of the melting point results which can not be explained by the uncertainty of temperature calibration or different sample purities. Only the extrapolated peak onset temperature $T_{e}$ is relatively independent of the above mentioned parameters and is therefore recommended to be used to characterize phase transitions [18]. The melting temperature results are categorized in accordance with the above mentioned temperature definitions. Temperatures which are not unambiguously defined in the research papers are either defined as temperature $T_{*}$ or, if possible, categorized based on the evaluation of the given heat flow signals. Qiu et al. [19] and Li et al. [20] have determined $T_{i}$ for the melting peak of octadecane. The majority of researchers have defined $T_{e}$ [21,22,23,24,25,26,27,28,29,30,31] or $T_{p}$ [19,20,29,31,32,33,34,35,36,37,38,39,40,41] as the melting temperature. Temperatures $T_{*}$ with insufficient information about their determination have been reported for DSC measurements [42,43,44,45,46,47,48,49,50,51], for adiabatic calorimeters (AC) [52,53,54,55] and for results which have been achieved with other not classified (NC) techniques [6,56,57,58,59,60,61,62]. A summary of all available data on the melting temperature can be found in Table 1 (alongside with data on the enthalpy, described in the next section) and Figure 1a which shows the melting temperatures arranged according to the given temperature definitions and its mean values.

### 2.2. Melting Enthalpy

As for the melting temperature, there are numerous results for the melting enthalpy of octadecane available in the literature. Many of these results were also obtained from the heat flow signal of DSC measurements, but without giving information about the way of determining the peak area which is the measure for the melting enthalpy [19,20,21,22,23,24,25,26,27,28,29,30,31,32,33,34,35,36,37,38,39,40,41,42,43,44,45,47,48,49,50,51]. The peak area depends on the integration limits and the type of the assumed baseline of the heat flow signal [18]. Therefore, beside the uncertainty of the measuring instruments itself, one reason for the spread of the melting enthalpy results can be the fact that researchers have applied different evaluation methods. Results achieved from AC have been reported from Schaerer et al. [52], Parks et al. [53], Messerly et al. [54] and Meyer and Meyer [55] and there is no information about the measuring principle for the data of Rossini [6]. A summary of all available data on the melting enthalpy can be found in Table 1 and Figure 1b which shows the available melting enthalpy results arranged in a histogram.

### 2.3. Density

**Figure 2:**
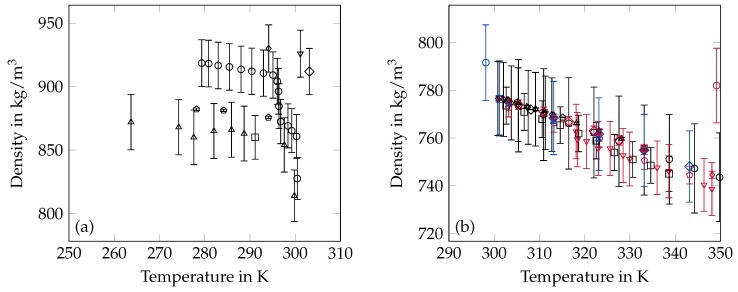
Summary of density data from the literature in the solid (**a**) and liquid (**b**) state. The error bars are the specified uncertainty of the data. The values named OR are results from our own research. (**a**) ∘ [62] □ [64] ⋄ [66] Δ [67] ∇ [73] ◊ [74] ⬠ OR; (**b**) ∘ [6] □ [27] ⋄ [27] Δ [28] ∇ [61] ◊ [62] ⬠ [63] ∘ [64] □ [65] ⋄ [67] Δ [68] ∇ [69] ◊ [70] ⬠ [71] ∘ [72] □ [73] ⋄ [75] Δ OR.

The available results of density measurements can be roughly categorized by the applied measuring principle. Pycnometers (PM) and dilatometers (DM) as independent measuring systems or combinations of both have been applied by the majority of researchers for liquid as well as solid state measurements [62,63,64,65,66,67,68,69]. Liquid state densities have additionally been determined by means of hydrometers (HM) [27,28] and vibrating-element systems (VE) [27,70]. Density measurements based on the method of a hydrostatic weighing (HW) have been performed by Graaf et al. [71] for the liquid state and in the framework of our own research (OR) for the liquid as well as the solid state. Furthermore, there are some publications with experimental results but without specification of the applied measuring principle [6,61,72,73] and the research of Müller and Lonsdale [74], who have applied X-ray measurements which are NC. A summary of all available data on the density can be found in Table 2 and Figure 2.

### 2.4. Heat Capacity

**Figure 3:**
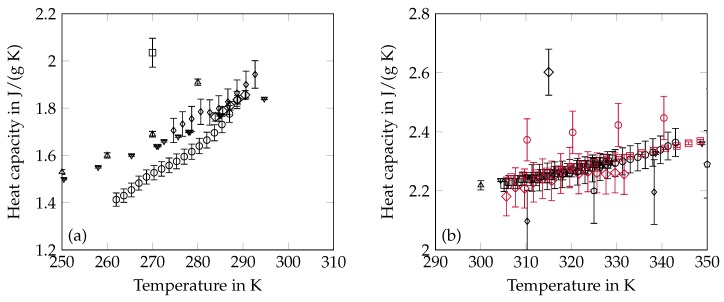
Summary of heat capacity data from the literature in the solid (**a**) and liquid (**b**) state. The error bars are the specified uncertainty of the data. The displayed data of Vélez et al. [27] and of our own research (OR) are reduced to every tenth and fourth point of the available results, respectively. (**a**) ∘ [27] □ [46] ⋄ [47] Δ [53] ∇ [54] ◊ OR; (**b**) ∘ [27] □ [45] ⋄ [46] Δ [53] ∇ [54] ◊ [67] ⬠ [76]∘ [77] □ [78] ⋄ OR.

The heat capacities are most commonly measured by means of DSC which can be operated with different methods. The dynamic mode works with a constant heating rate and has been applied by Höhne [76], Djordjevic and Laub [46], Durupt et al. [77], Fonseca et al. [47] and Vélez et al. [27]. The step mode applies discrete temperature steps to the sample and the heat flow is determined for each temperature step [45]. Dynamic measurements have been conducted in the framework of our own research and the results are included as well. Some researchers have measured the specific heat capacity with AC [53,54,78] and there is no information about the measuring principle for the data of Shlosinger and Bentilla [67]. A summary of all available data on the heat capacity can be found in Table 3 and Figure 3.

### 2.5. Thermal Conductivity

**Figure 4:**
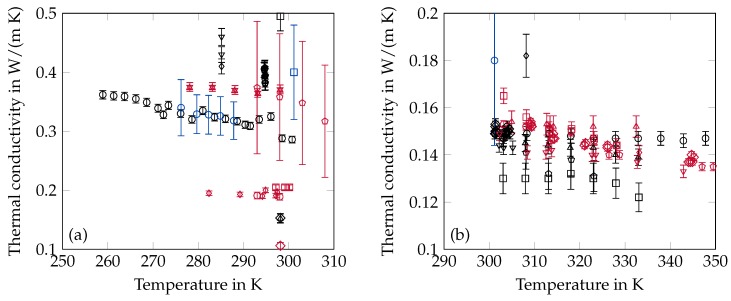
Summary of thermal conductivity data from the literature in the solid (**a**) and liquid (**b**) state. The error bars are the specified uncertainty of the data. (**a**) ∘ [27] □ [32] ⋄ [38] Δ [39] ∇ [80] ◊ [80] ⬠ [80] ∘ [81] □ [82] ⋄ [83] Δ [85] ∇ [86] ◊ [88] ⬠ [90] ∘ [91] □ [94]; (**b**) ∘ [27]□ [28] ⋄ [80] Δ [81] ∇ [82] ◊ [83] ⬠ [84] ∘ [85] □ [86] ⋄ [87] Δ [88] ∇ [89] ◊ [92] ⬠ [93] ∘ [94].

Thermal conductivities have been measured with transient as well as stationary measurement methods. Irby et al. [80], Harish et al. [81], Wu et al. [82], Vélez et al. [27], Khadiran et al. [83] and Águila V et al. [84] applied the transient hot wire (TW) method and Jeon et al. [32], Yu et al. [38], Motahar et al. [85] and Motahar et al. [86] used a transient plane source (TP) to measure solid and liquid state thermal conductivities while Ho and Gao [28] have measured the liquid state only with a transient thermal analyser (TA). The stationary methods can be categorized in measurement set-ups analysing the heat flow between parallel plates (SP, stationary plate) [87,88] or coaxial cylinder systems (SC, stationary cylinder) [89,90,91]. Irby et al. [80] have achieved some additional results by applying inverse methods (IM) like phase change- and transient conduction experiments. Thermal conductivity measurements which can not be classified according to the above mentioned categories have been performed by Mustafaev [92], Rastorguev and Bogatov [93] and Holmen et al. [94]. A summary of all available data on the thermal conductivity can be found in Table 4 and Figure 4.

### 2.6. Viscosity

**Figure 5:**
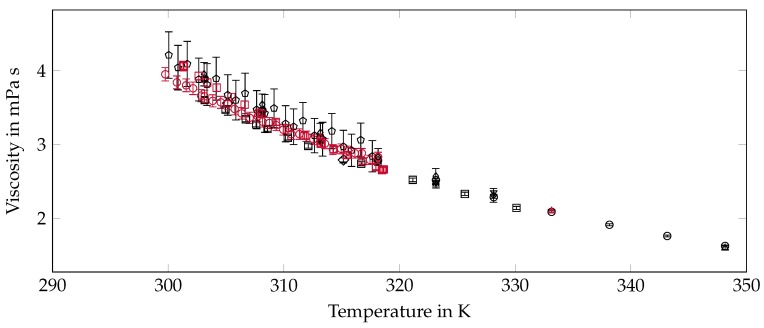
Summary of viscosity data from the literature. The error bars are the specified uncertainty of the data. The displayed data of Delgado et al. [98] are reduced to every fifth point of the available results. ∘ [6] □ [28] ⋄ [63] Δ [70] ∇ [84] ◊ [85] ⬠ [98] ∘ [98] □ [98] ⋄ [99] Δ [100].

The majority of researchers have determined the viscosity of octadecane with rotational rheometers (RR) [28,84,85,98]. Results of our own measurements with a translational rheometer (TR), the so called IMETER, are included in the publication of Delgado et al. [98]. Hogenboom et al. [99] and Ducoulombier et al. [100] have applied falling-body viscometers (FV) and Dover and Hensley [63] used an Ostwald capillary viscometer (CV). A self-built vibrating-wire viscometer (VV) has been designed for the measurements of Caudwell et al. [70] and there is no information about the measuring principle for the data of Rossini [6]. A summary of all available data on the viscosity can be found in Table 5 and Figure 5.

## 3. Preselection of Data

The results presented in Section 5 are obtained after a preselection of the literature values by excluding obvious outliers and less appropriate data. The reasons for the exclusion are the following:

### 3.1. Melting Temperature and Enthalpy

- Only temperature data which were achieved as the extrapolated onset temperature $T_{e}$ or the temperature $T_{*}$ (see Section 2.1) are considered since these temperatures seem to represent a realistic melting point of octadecane.
- Jeong et al. [23] and Qiu et al. [31] were removed because they were outliers on the high and low side of temperature data in the literature.
- Boudouh et al. [48], Babich et al. [40] and Zhang et al. [24] were removed because they were outliers on the high and low side of literature melting enthalpy data.
- The data of He et al. [37], Zhang et al. [39] and Zhu et al. [41] were excluded since these authors investigated octadecane of low purity.
- Yu et al. [38] were not considered due to identical melting point and enthalpy results compared to a previous publication of the same co-authors [37] despite supposedly different purity grades of octadecane.

### 3.2. Density

- One datapoint of Shlosinger and Bentilla [67] was neglected since it was located in the 2-phase-region.
- The whole series of solid state data from Seyer et al. [62] was removed because of the indicated solid-solid transformation.
- All data of Würflinger and Schneider [73] were excluded due to the applied inverse method for determining the solid-state density.
- The data of Müller and Lonsdale [74] were neglected since their results where achieved with X-ray measurements resulting in very high theoretical density calculations based on the distance between the molecules.
- Liquid state densities from van Hook and Silver [64] were excluded because of incomprehensible corrections in their data.
- The liquid state data point of McKinney [72] was removed since it was given at a temperature of 25 ${}^{\circ }$C which is obviously in the solid state.

### 3.3. Thermal Conductivity

- The thermal conductivity data of Harish et al. [81] were not considered because they applied octadecane itself for calibrating their measuring system.
- Solid state data points of Jeon et al. [32] and Khadiran et al. [83] were removed because they are outliers on the high and low side of data in the literature.
- Yu et al. [38] and Zhang et al. [39] were excluded because of the ambiguous specifications of the evaluation temperature (at room temperature...).
- Two data point of Griggs and Yarbrough [90] were removed from the solid state data due to evaluation temperatures above the melting point.
- Liquid state data of Holmen et al. [94] and Khadiran et al. [83] were neglected since they were significantly higher than the remaining data points.

### 3.4. Heat Capacity

- Data points near the phase change temperature were neglected since they may be affected by phase change phenomena and therefore do not describe pure sensible heating of octadecane.
- The liquid state heat capacity of Parks et al. [53] was excluded because the indicated temperature is in the solid state range.
- The data of Djordjevic and Laub [46] were removed in both phases because they were outliers on the high side of values found in the literature.

### 3.5. Viscosity

The viscosity data of Hogenboom et al. [99] were not considered because they were measured at higher temperatures out of the range of interest for our study.

## 4. Statistics

For all properties, we investigated the relationship with temperature and chose the specification with the highest polynomial of temperature that was found to be statistically significant, i.e., for the corresponding parameter of which we could reject the null hypothesis $H_{0}:\beta_{k}=0$ in a two-sided test, where $\beta_{k}$ denotes the parameters. Except for the viscosity, where we followed the recommendation of the VDI heat atlas [101]:

(1) $$ln\left(\eta \right)=A+\frac{B}{T}$$

All of these relationships are linear in parameters and hence we fitted specifications of the following form:

(2) $$y_{i}=x_{i}^{\prime }\beta +\varepsilon_{i},$$

were ${}^{\prime }$ is the transpose of a vector and therefore $x_{i}^{\prime }\beta$ is the scalar product. The index *i* denotes the observation, $y_{i}$ in turn represents the measured values of (solid and liquid) density, (solid and liquid) heat capacity, (solid and liquid) thermal conductivity, and the natural logarithm of viscosity. The vector β denotes the corresponding true (unobserved) parameter vector and ε is a random error term that is potentially correlated for different observations from the same study. In the most common case, we ended up fitting a linear relationship and $x_{i}$ hence comprises a constant and the temperature $T_{i}$ at which the measurement was obtained (i.e., $x_{i}={\left[1\;T_{i}\right]}^{\prime }$). Specifically, we fitted this simple linear model for the liquid density and the solid and liquid thermal conductivity. In the solid density and the solid thermal conductivity case, we found no significant evidence for a relationship with temperature and therefore only fitted a constant (i.e., $x_{i}=1$). For liquid heat capacity, we found a significant quadratic relationship with temperature and hence fitted a second-order polynomial (i.e., $x_{i}={\left[1\;T_{i}\;{\left(T_{i}\right)}^{2}\right]}^{\prime }$). Finally, for the viscosity, we fitted a constant and a linear relationship with the inverse temperature (i.e., $x_{i}={\left[1\;{\left(T_{i}\right)}^{-1}\right]}^{\prime }$).

Besides the data points ($y_{i},T_{i}$) themselves, we have information on the corresponding uncertainty in the measurement of *y*. If this reported uncertainty actually represents a good approximation to the relative size of the unsystematic component $\varepsilon_{i}$ in (2) across observations, it can be used to weight observations in the estimation in order to obtain more precise estimates of β. For the solid density and solid heat conductivity, we expect the measurement error to be of minor magnitude compared to variation around the systematic relationship that is introduced e.g., by differences in the physical sample preparation. Further, for viscosity, the uncertainty refers to the level while we fit a linear model to the natural logarithm. In these three cases, we therefore decided not to use the provided uncertainty information in the corresponding regressions and used the ordinary least squares (OLS) estimator:

(3) $${\hat{\beta }}_{OLS}={\left(X^{\prime }X\right)}^{-1}X^{\prime }y,$$

where $X={\left[x_{1}\;x_{2}\;\cdots \;x_{N}\right]}^{\prime }$, $y={\left[y_{1}\;y_{2}\;\cdots \;y_{N}\right]}^{\prime }$, and *N* denotes the number of observations. Note that—as mentioned above—we did not find a significant effect of temperature for two of these cases and therefore finally only fitted a constant, in which case (3) reduces to taking the mean of *y*.

For all other properties, we expect the provided uncertainties to capture the magnitude of the unsystematic variation associated with different observations of the same property well and therefore estimated the corresponding parameter vectors β with a weighted least squares (WLS) procedure. Specifically, denoting the uncertainty of an observation by σ, the estimates are obtained using the following estimator:

(4) $${\hat{\beta }}_{WLS}={\left(X^{\prime }WX\right)}^{-1}X^{\prime }Wy,$$

where W is an N×N diagonal weighting matrix with $w_{ii}=1/\sigma^{2}$.

**Figure 6:**
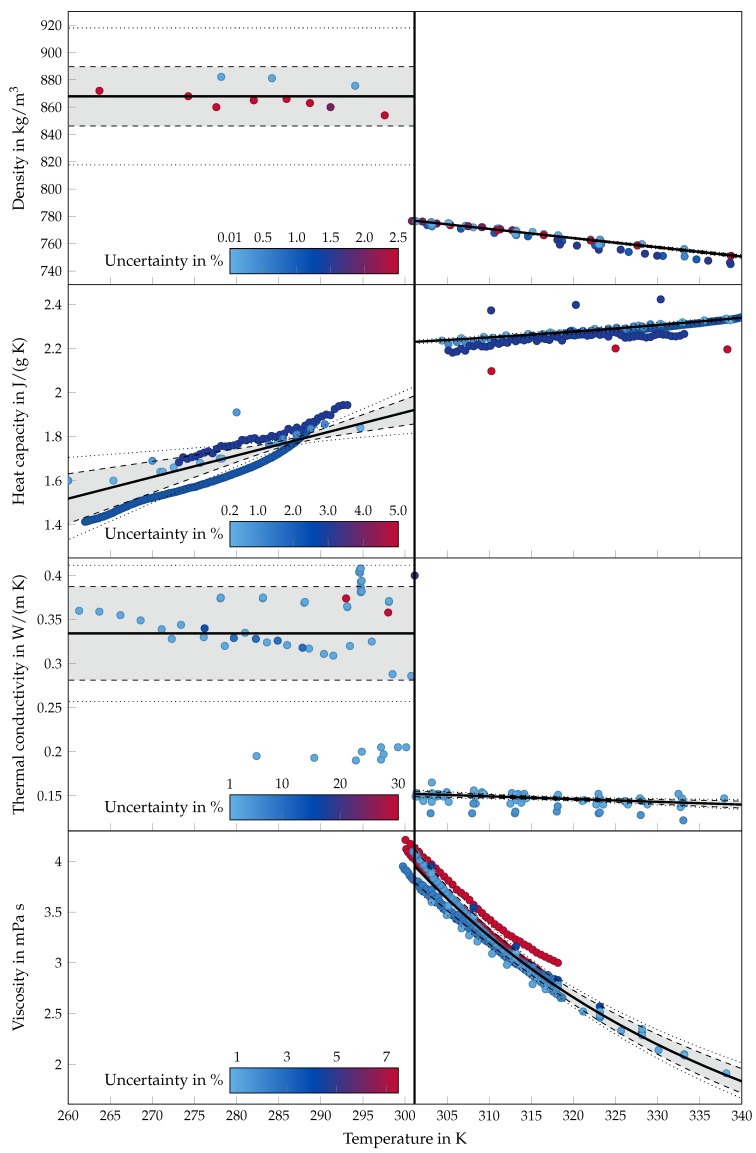
Summary of the estimated fit functions for the temperature-dependent thermophysical properties of octadecane. The displayed points indicate the preselected data applied for the calculation and the color scale corresponds to the associated uncertainty. The grey shaded areas between the dashed lines describe the confidence interval of 95% and the dotted lines a confidence of 99%.

With an estimated parameter vector $\hat{\beta }$ at hand, we can calculate fitted values:

(5) $$\hat{y}=x^{\prime }\hat{\beta },$$

where x can be an actual data point or any point at which we are interested in the predicted physical property. Fitted values are depicted by the solid lines in Figure 6 in Section 5.

In assessing the precision with which we estimated $\hat{\beta }$ (and hence $\hat{y}$), we allow errors of different observations from the same study to be correlated by relying on cluster-robust inference. Specifically, denoting the different studies/clusters by g=1,2,…,G, the estimated variance-covariance matrix of the estimated parameter vectors ${\hat{\beta }}_{OLS}$ and ${\hat{\beta }}_{WLS}$ are given by:

(6) $${\hat{V}}_{OLS}={\left(X^{\prime }X\right)}^{-1}\left(\sum_{g=1}^{G}X_{g}^{\prime }{\tilde{\varepsilon }}_{g}{\tilde{\varepsilon }}_{g}^{\prime }X_{g}\right){\left(X^{\prime }X\right)}^{-1}$$

and

(7) $${\hat{V}}_{WLS}={\left(X^{\prime }WX\right)}^{-1}\left(\sum_{g=1}^{G}X_{g}^{\prime }W_{g}{\tilde{\varepsilon }}_{g}{\tilde{\varepsilon }}_{g}^{\prime }W_{g}X_{g}\right){\left(X^{\prime }WX\right)}^{-1},$$

where ${\tilde{\varepsilon }}_{g}=\sqrt{\frac{G}{G-1}\frac{N-1}{N-K}}\left(y_{g}-X_{g}^{\prime }\hat{\beta }\right)$ is the vector of residuals (multiplied with a correction factor for small numbers of clusters) and the *g* subscript indicates that only the elements of the corresponding matrices or vectors that belong to cluster *g* are considered (see Cameron and Miller [102] for details on cluster-robust standard errors).

Given the fitted values and an estimate of the variance-covariance matrix, we can construct (1−α)×100% confidence intervals for all estimated relationships as follows:

(8) $$\hat{y}\pm t_{\alpha /2}\sqrt{x^{\prime }\hat{V}x}$$

where $t_{\alpha /2}$ is the critical value from a t-distribution with G−1 degrees of freedom for a significance level α (i.e., $P\left(t<-t_{\alpha /2}\right)+P\left(t>t_{\alpha /2}\right)=1-\alpha$). The 95% and 99% confidence intervals are depicted in Figure 6 as dashed and dotted lines, respectively. For the viscosity, we display the exponential of both the fitted values and the confidence bounds.

All statistical analyses were performed using Stata (version 15.1 MP).

## 5. Results and Discussion

Our statistical analysis reveals a clear difference between the confidence that we can put into the fit functions that describe the solid and liquid material properties of octadecane (Figure 6). All fit functions for the liquid state are determined with small confidence intervals. In contrast, two of the three solid state fit functions are highly uncertain, namely the ones describing the solid thermal conductivity and the solid density. In the first case, there are enough data points but their distribution is broad and without a recognizable trend. Also remarkable is the accumulation of points around a low value of 0.2 W/(m K), which were measured by different authors. Therefore, the confidence interval surrounding the mean is wide and no temperature dependence of the solid thermal conductivity could be determined with statistical significance. In the second case, the statistical analysis is constrained by the small number of available data points respectively the small number of studies which measured the solid density. Here, too no temperature dependency could be determined with statistical significance. Moreover, the 95% confidence interval is wider than the range of the data points. We assume that the reasons for the scattered solid values is sample preparation (degassing and cooling rate) and experimental procedure, which is especially important for solids [103].

Furthermore, a few values for the viscosity and the liquid density lie in the solid phase, which is defined by our mean melt temperature. The reasons for this are uncertainties in the temperature measurements.

Table 6 summarizes the estimated parameters of the fit functions for the solid and the liquid state. The fit functions are valid from the mean melting point to ±40 K. Also included in the table are the mean melting point (301.13 K) and the mean melting enthalpy (236.98 J/g). The functions for the indicated confidence intervals are summarized in the Appendix (Equations (A1)–(A18)).

**Figure A1:**
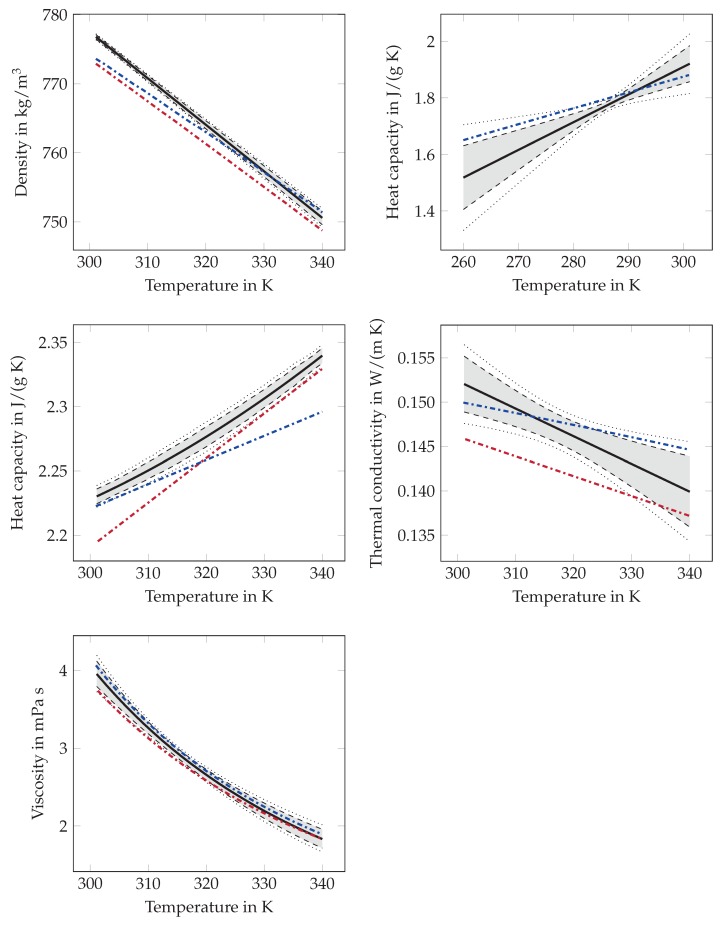
Comparison of the estimated fit functions with functions from literature. The black lines are the estimated fit functions from Table 6 (solid line) and the corresponding confidence intervals of 95 % (dashed line) and 99 % (dotted line) from Appendix A.1. The red and blue lines represent the functions from the VDI heat atlas [101] and from Yaws [104], respectively.

A comparison with existing functions for the liquid state properties of octadecane from the VDI heat atlas [101] and from Yaws [104] shows satisfactory agreement with the determined fit functions in the temperature interval under consideration. The maximum relative deviations between estimated fit functions and the functions are 0.5 %, 1.9 %, 4 % and 5 % for the liquid state density, heat capacity, thermal conductivity and viscosity, respectively. For the solid state properties there is only one function available from Yaws [104] for the heat capacity which shows a maximum relative deviation of 9 %. A graphical overview of the functions can be found in Figure A1 in Appendix A.2.

## 6. Conclusions

The temperature-dependent functions of the thermophysical properties of octadecane derived in this review paper can be used for numerical and/or analytical calculations. The usage of these functions improves the comparability of studies and simplifies validation. The given confidence intervals help to estimate the accuracy of the results. Generally, the confidence intervals around the liquid functions are considerably thinner than around the solid functions. The two properties which are most insecure are the solid thermal conductivity and the solid density. In our opinion, these two quantities are both greatly affected by sample preparation in the experiments. We therefore hope that this research leads to further measurements with a standardized measurement protocol. 

## Figures and Tables

**Table 1:** Summary of melting temperature and enthalpy data from the literature.

Reference	Purity in %	Year	Method	Temperature in K	Uncert. in K	Enthalpy in J/g	Uncert. in %
Rossini [6]	n/a	1952	NC	$T_{*}$ = 301.34	0.02	243.6	0.3
Qiu et al. [19]	99	2012	DSC, 5 K/min	$T_{i}$ = 298.65	n/a	223.1	n/a
				$T_{p}$ = 301.55	n/a		
				$T_{f}$ = 303.65	n/a		
Li et al. [20]	99	2011	DSC, 10 K/min	$T_{i}$ = 298.36	n/a	235.9	n/a
				$T_{p}$ = 302.97	n/a		
Tang et al. [21]	97	2017	DSC, 5 K/min	$T_{e}$ = 301.68 ${}^{a}$	0.2	239.32	5
Bayramoglu [22]	100	2011	DSC, 10 K/min	$T_{e}$ = 301.11 ${}^{a}$	n/a	239.89	n/a
Jeong et al. [23]	n/a	2013	DSC, 5 K/min	$T_{e}$ = 303.55	n/a	247.6 ${}^{b}$	n/a
Zhang et al. [24]	n/a	2013	DSC, 5 K/min	$T_{e}$ = 299.99 ${}^{a}$	n/a	207.2	n/a
Sun et al. [25]	n/a	2013	DSC, 10 K/min	$T_{e}$ = 301.04	n/a	218.8	n/a
Wang and Lu [26]	99	2013	DSC, 0.5-1.5 K/min	$T_{e}$ = 301.55	n/a	230.5	n/a
Vélez et al. [27]	99	2015	DSC, 2 K/min	$T_{e}$ = 300.22 ${}^{a}$	0.095	243.68	0.04
Ho and Gao [28]	99.9	2009	DSC, 2 K/min	$T_{e}$ = 299.65 ${}^{a}$	n/a	243.1	n/a
Li et al. [29]	97	2010	DSC, 5 K/min	$T_{e}$ = 301.85 ${}^{a}$	0.2	232.49	5
				$T_{p}$ = 303.47	0.2		
Döğüşcü et al. [30]	n/a	2018	DSC, 3 K/min	$T_{e}$ = 300.95	n/a	226.2	n/a
Qiu et al. [31]	99	2015	DSC, 5 K/min	$T_{e}$ = 298.65	n/a	227.1	n/a
				$T_{p}$ = 301.55	n/a		
Jeon et al. [32]	n/a	2012	DSC, 5 K/min	$T_{p}$ = 302.06 ${}^{a}$	n/a	241.97	n/a
Zhang et al. [33]	99.9	2012	DSC, 0.2 K/min	$T_{p}$ = 303.25 ${}^{a}$	n/a	220.4	n/a
Shan et al. [34]	95	2009	DSC, 10 K/min	$T_{p}$ = 304.15 ${}^{a}$	n/a	222	n/a
Chaiyasat et al. [35]	99.5	2012	DSC, 5 K/min	$T_{p}$ = 303.15 ${}^{a}$	n/a	241.7	n/a
Chung et al. [36]	n/a	2015	DSC, 10 K/min	$T_{p}$ = 301.85 ${}^{a}$	n/a	226	n/a
He et al. [37]	90	2014	DSC, 10 K/min	$T_{p}$ = 301.89 ${}^{a}$	n/a	209.1	n/a
Yu et al. [38]	98.5	2014	DSC, 10 K/min	$T_{p}$ = 301.89 ${}^{a}$	n/a	209.1	n/a
Zhang et al. [39]	90	2012	DSC, 10 K/min	$T_{p}$ = 301.25 ${}^{a}$	n/a	212.6	n/a
Babich et al. [40]	n/a	1992	DSC, 2 K/min	$T_{p}$ = 301.6	n/a	200	n/a
Zhu et al. [41]	90	2016	DSC, 10 K/min	$T_{p}$ = 301.7 ${}^{a}$	n/a	204.4	6
Wei et al. [42]	99	2014	DSC, 1 K/min	$T_{*}$ = 300.95	0.2	242.24	1
Chang et al. [43]	97	1983	DSC, 5 K/min	$T_{*}$ = 301.1	n/a	233.4	n/a
Kolesnikov and Syunyaev [44]	n/a	1985	DSC, 8 and 1 K/min	$T_{*}$ = 301.00	n/a	238.7	n/a
Huang et al. [45]	99	2005	DSC	$T_{*}$ = 300.83	n/a	232.3	n/a
Djordjevic and Laub [46]	n/a	1986	DSC	$T_{*}$ = 301.6	n/a		
Fonseca et al. [47]	99.5	2014	DSC, 0.48 K/min	$T_{*}$ = 301.46	0.1	241.02	1
Boudouh et al. [48]	99	2016	DSC, 0.8 K/min	$T_{*}$ = 300.3	0.1	256.7	0.5
Mondieig et al. [49]	99	2004	DSC, 2 K/min	$T_{*}$ = 301.1	n/a	236.1	n/a
Robles et al. [50]	99.4	1996	DSC, 2 K/min	$T_{*}$ = 301.1	0.6	235.0	7
Wei et al. [51]	99.5	2013	DSC, 1 K/min	$T_{*}$ = 300.9	0.2	242.2	1
Schaerer et al. [52]	99.9	1955	AC	$T_{*}$ = 301.35	n/a	241.3	n/a
Parks et al. [53]	96	1949	AC	$T_{*}$ = 301.3	n/a	237.8	n/a
Messerly et al. [54]	99.98	1967	AC	$T_{*}$ = 301.33	n/a	242.5	n/a
Meyer and Meyer [55]	99.9	1983	AC	$T_{*}$ = 301.27	n/a	236.5	n/a
Ksiazczak [56]	99.7	1989	NC	$T_{*}$ = 301.27	0.02		
Carey and Smith [57]	97	1933	NC	$T_{*}$ = 300.85	n/a		
Domańska et al. [58]	n/a	1999	NC	$T_{*}$ = 301.65	n/a		
Levene et al. [59]	n/a	1915	NC	$T_{*}$ = 301.15	n/a		
Parks et al. [60]	95	1946	NC	$T_{*}$ = 300.85	n/a		
Krafft [61]	n/a	1882	NC	$T_{*}$ = 301.15	n/a		
Seyer et al. [62]	n/a	1944	NC	$T_{*}$ = 301.25	n/a		

${}^{a}$ Determined from plot; ${}^{b}$ Between 28–32 °C.

**Table 2:** Summary of density data from the literature.

Reference	Purity in %	Year	Method	Uncertainty in %	Observations	
					Solid	Liquid
Rossini [6]	n/a	1944	NC	2.5	0	52
Vélez et al. [27]	99	2015	HM/VE	1/0.01	0	10/2
Ho and Gao [28]	99.9	2009	HM	0.07	0	10
Krafft [61]	n/a	1882	NC	2 ${}^{a}$	0	1
Seyer et al. [62]	n/a	1944	DM	2 ${}^{a}$	16	8
Dover and Hensley [63]	n/a	1934	PM	0.02 ${}^{b}$	0	2
van Hook and Silver [64]	99	1942	DM	2 ${}^{a}$	1	1
Cutler et al. [65]	high purity	1958	DM	0.1	0	5
Nelson et al. [66]	n/a	1960	DM	2 ${}^{a}$	1	0
Shlosinger and Bentilla [67]	n/a	1965	PM	2.5 (s)/0.26 (l)	8	6
Findenegg [68]	99	1970	PM	0.02	0	6
Espeau and Céolin [69]	n/a	2006	PM and DM	1.5	0	99
Caudwell et al. [70]	99	2004	VE	0.20	0	7
Graaf et al. [71]	n/a	1992	HW	0.5	0	10
McKinney [72]	n/a	1923	NC	2 ${}^{a}$	0	1
Würflinger and Schneider [73]	99	1973	NC	2 ${}^{a}$	1	1
Müller and Lonsdale [74]	n/a	1948	NC	2 ${}^{a}$	1	0
Dutour et al. [75]	99	2000	VE	2 ${}^{a}$	0	8
Own research (OR)	97	2018	HW	0.1	3	3

${}^{a}$ Assumption, ${}^{b}$ Assumed value from Findenegg [68].

**Table 3:** Summary of heat capacity data from the literature. The number behind the abbreviation DSC describes either the heating rate (K/min) or the step size (K) of the applied measurement method.

Reference	Purity in %	Year	Method	Uncertainty in %	Observations	
					Solid	Liquid
Vélez et al. [27]	99	2015	DSC, 5 K/min	2 ${}^{a}$	166	198
Huang et al. [45]	99	2005	DSC, 1 K	1	0	25
Djordjevic and Laub [46]	n/a	1983	DSC, 5 K/min	3 ${}^{a}$	1	1
Fonseca et al. [47]	99.5	2014	DSC, 0.48 K/min	1	5	0
Parks et al. [53]	96	1949	AC	0.7	21	1
Messerly et al. [54]	99.98 (mol)	1967	AC	0.2	77	11
Shlosinger and Bentilla [67]	n/a	1965	NC	5 ${}^{b}$	0	5
Höhne [76]	very pure	1981	DSC, 10 K/min	5	0	3
Durupt et al. [77]	99	1996	DSC	3 ${}^{a}$	0	9
van Miltenburg [78]	99.8	1999	AC	0.2	0	38
Own research (OR)	97	2018	DSC, 1 K/min	3	41	57

${}^{a}$ Taken from Czichos et al. [79], ${}^{b}$ Assumption.

**Table 4:** Summary of thermal conductivity data from the literature.

Reference	Purity in %	Year	Method	Uncertainty in %	Observations	
					Solid	Liquid
Vélez et al. [27]	99	2015	TW	2	20	10
Ho and Gao [28]	99.9	2009	TA	5	0	7
Jeon et al. [32] ${}^{a}$	n/a	2012	TP	5 ${}^{b}$	1	0
Yu et al. [38] ${}^{a}$	98.5	2014	TP	5 ${}^{c}$	1	0
Zhang et al. [39] ${}^{a}$	90	2012	TP	5 ${}^{d}$	1	0
Irby et al. [80]	n/a	1988	TW/IM	1.5-3	15/3	13
Harish et al. [81] ^e^	n/a	2015	TW	3	2	7
Wu et al. [82]	99	2015	TW	2	3	3
Khadiran et al. [83]	n/a	2015	TW	5 ${}^{f}$	1	1
Águila V et al. [84]	99	2018	TW	5	0	4
Motahar et al. [85]	99	2014	TP	1	5	6
Motahar et al. [86]	99	2016	TP	2	5	6
Sakiadis and Coates [87]	95	1957	SP	1	0	17
Powell et al. [88]	n/a	1961	SP	2 ${}^{g}$	6	6
Ziebland and Patient [89]	n/a	1962	SC	2	0	8
Griggs and Yarbrough [90]	99	1978	SC	30	4	0
Yarbrough and Kuan [91]	n/a	1981	SC	10-14	5	0
Mustafaev [92]	n/a	1973	NC	2	0	4
Rastorguev and Bogatov [93]	n/a	1972	NC	1.3 ${}^{h}$	0	4
Holmen et al. [94]	99	2002	NC	20	1	1

${}^{a}$ No information about temperature given; 298.15 K assumed; ${}^{b}$ From manufacturers data sheet [95]; ${}^{c}$ From manufacturers data sheet [96]; ${}^{d}$ Assumption; ^e^ Octadecane has been applied for calibration purpose; ${}^{f}$ Taken from Águila V et al. [84]; ${}^{g}$ Taken from Czichos et al. [79]; ${}^{h}$ Taken from Rastorguev et al. [97];

**Table 5:** Summary of viscosity data from the literature.

Reference	Purity in %	Year	Method	Uncertainty in %	Observations Liquid
Rossini [6]	n/a	1952	NC	0.7	58
Ho and Gao [28]	99.9	2009	RR	1	11
Dover and Hensley [63]	n/a	1934	CV	1 ${}^{a}$	2
Caudwell et al. [70]	99	2004	VV	2	7
Águila V et al. [84]	99	2018	RR	1	6
Motahar et al. [85]	99	2014	RR	4	6
Delgado et al. [98] ${}^{b}$	97	2018	TR/RR/RR	1.38/7.74/2.27	103/110 ${}^{c}$/148 ${}^{c}$
Hogenboom et al. [99]	high purity	1967	FV	5	3
Ducoulombier et al. [100]	purum	1986	FV	1 ${}^{d}$	4

${}^{a}$ Assumption; ${}^{b}$ The listed data are for the IMETER/Anton Paar MCR502/TA Instruments AR G2. ${}^{c}$ The data points are acquired for two different operating modes (rotational and oscillatory). ${}^{d}$ Uncertainty of the falling time of the falling body only.

**Table 6:** Estimated fit functions for the temperature-dependent thermophysical properties of octadecane and its mean melting temperature and enthalpy.

Property	Solid State		Liquid State
	(261.13 K–301.13 K)		(301.13 K–341.13 K)
Density in kg/m${}^{3}$	867.914		979.826−0.674·T
Heat capacity in J/(g K)	$-1.029+9.797\cdot 10^{-3}\cdot T$		$3.247-8.861\cdot 10^{-3}\cdot T+1.821\cdot 10^{-5}\cdot T^{2}$
Thermal conductivity in W/(m K)	0.334		$0.246-3.121\cdot 10^{-4}\cdot T$
Viscosity in mPa s	-		$exp\left(-5.353+2026.013/T\right)$
Melting temperature in K	-	301.13	-
Melting enthalpy in J/g	-	236.98	-

## Abbreviations

The following abbreviations are used in this manuscript:

A	Coefficient
B	Coefficient
AC	Adiabatic calorimeter
CV	Capillary viscometer
DM	Dilatometer
DSC	Differential scanning calorimetry
FV	Falling-body viscometer
*g*	Study/cluster number
*G*	Total number of studies/clusters
HM	Hydrometer
HW	Hydrostatic weighing
$H_{0}$	Null hypothesis
IM	Inverse method
*N*	Number of observations
n/a	Not available
NC	Not classified
OR	Own research
PM	Pycnometer
RR	Rotational rheometer
SC	Stationary cylinder
SP	Stationary plate
$t_{\alpha /2}$	Critical value from a student t-distribution
*T*	Temperature
TA	Transient thermal analyser
$T_{c}$	Extrapolated offset temperature
$T_{e}$	Extrapolated onset temperature
$T_{f}$	Final peak temperature
$T_{i}$	Initial peak temperature
$T_{p}$	Peak maximum temperature
TP	Transient plane source
$T_{*}$	Temperature with insufficient information about its determination
TR	Translational rheometer
TW	Transient hot wire
VE	Vibrating-element
VV	Vibrating viscometer
${\hat{V}}_{OLS}$	Esitmated variance-covariance matrix of the ordinary least squares estimator
${\hat{V}}_{WLS}$	Esitmated variance-covariance matrix of the weighted least squares estimator
W	Weighting matrix
x	Explanantory variable vector
X	Explanantory variable matrix
*y*	Measured values
y	Vector of measured values
$\hat{y}$	Estimated value
α	Significance level
β	Parameter
β	Parameter vector
$\hat{\beta }$	Estimated parameter vector
${\hat{\beta }}_{OLS}$	Ordinary least squares estimator
${\hat{\beta }}_{WLS}$	Weighted least squares estimator
ε	Random error term
$\tilde{\varepsilon }$	Vector of residuals
η	Viscosity
σ	Uncertainty of observation

## Appendix A

### Appendix A.1. Estimated Fit Functions and Confidence Intervals

#### Appendix A.1.1. Melting Temperature

Functions for the confidence interval of 95%:

(A1) $$\begin{array}{c} \max/\min=301.1327586\pm 0.164303685 \end{array}$$

Functions for the confidence interval of 99%:

(A2) $$\begin{array}{c} \max/\min=301.1327586\pm 0.221642561 \end{array}$$

#### Appendix A.1.2. Melting Enthalpy

Functions for the confidence interval of 95%:

(A3) $$\begin{array}{c} \max/\min=236.9828571\pm 2.901537323 \end{array}$$

Functions for the confidence interval of 99%:

(A4) $$\begin{array}{c} \max/\min=236.9828571\pm 3.957815931 \end{array}$$

#### Appendix A.1.3. Density

Functions for the confidence interval of 95%, solid state:

(A5) $$\begin{array}{c} \max/\min=867.9136364\pm 21.74247525 \end{array}$$

Functions for the confidence interval of 99%, solid state:

(A6) $$\begin{array}{c} \max/\min=867.9136364\pm 50.15293385 \end{array}$$

Functions for the confidence interval of 95%, liquid state:

(A7) $$\begin{array}{c} \begin{array}{c} \max/\min=979.8260505-0.674202476\cdot T\pm 2.131449546\cdot (9.778011011+\\ -0.065513473\cdot T+0.000109976\cdot T^{2}{)}^{1/2} \end{array} \end{array}$$

Functions for the confidence interval of 99%, liquid state:

(A8) $$\begin{array}{c} \begin{array}{c} \max/\min=979.8260505-0.674202476\cdot T\pm 2.946712883\cdot (9.778011011+\\ -0.065513473\cdot T+0.000109976\cdot T^{2}{)}^{1/2} \end{array} \end{array}$$

#### Appendix A.1.4. Heat Capacity

Functions for the confidence interval of 95%, solid state:

(A9) $$\begin{array}{c} \begin{array}{c} \max/\min=-1.029094213+0.009797222\cdot T\pm 2.776445105\cdot (0.191470758+\\ -0.001336832\cdot T+2.33367\cdot 10^{-6}\cdot T^{2}{)}^{1/2} \end{array} \end{array}$$

Functions for the confidence interval of 99%, solid state:

(A10) $$\begin{array}{c} \begin{array}{c} \max/\min=-1.029094213+0.009797222\cdot T\pm 4.604094871\cdot (0.191470758+\\ -0.001336832\cdot T+2.33367\cdot 10^{-6}\cdot T^{2}{)}^{1/2} \end{array} \end{array}$$

Functions for the confidence interval of 95%, liquid state:

(A11) $$\begin{array}{c} \begin{array}{c} \max/\min=3.246741729-0.008860513\cdot T+1.82147\cdot 10^{-5}\cdot T^{2}\pm 2.364624252\cdot (0.407699103+\\ -0.005073903\cdot T+2.36524\cdot 10^{-5}\cdot T^{2}-4.89464\cdot 10^{-8}\cdot T^{3}+3.79394\cdot 10^{-11}\cdot T^{4}{)}^{1/2} \end{array} \end{array}$$

Functions for the confidence interval of 99%, liquid state:

(A12) $$\begin{array}{c} \begin{array}{c} \max/\min=3.246741729-0.008860513\cdot T+1.82147\cdot 10^{-5}\cdot T^{2}\pm 3.499483297\cdot (0.407699103+\\ -0.005073903\cdot T+2.36524\cdot 10^{-5}\cdot T^{2}-4.89464\cdot 10^{-8}\cdot T^{3}+3.79394\cdot 10^{-11}\cdot T^{4}{)}^{1/2} \end{array} \end{array}$$

#### Appendix A.1.5. Thermal Conductivity

Functions for the confidence interval of 95%, solid state:

(A13) $$\begin{array}{c} \max/\min=0.334295082\pm 0.053078735 \end{array}$$

Functions for the confidence interval of 99%, solid state:

(A14) $$\begin{array}{c} \max/\min=0.334295082\pm 0.077233044 \end{array}$$

Functions for the confidence interval of 95%, liquid state:

(A15) $$\begin{array}{c} \begin{array}{c} \max/\min=0.246046459-0.000312171\cdot T\pm 2.20098516\cdot (0.000557934+\\ -3.50915\cdot 10^{-6}\cdot T+5.52305\cdot 10^{-9}\cdot T^{2}{)}^{1/2} \end{array} \end{array}$$

Functions for the confidence interval of 99%, liquid state:

(A16) $$\begin{array}{c} \begin{array}{c} \max/\min=0.246046459-0.000312171\cdot T\pm 3.105806516\cdot (0.000557934+\\ -3.50915\cdot 10^{-6}\cdot T+5.52305\cdot 10^{-9}\cdot T^{2}{)}^{1/2} \end{array} \end{array}$$

#### Appendix A.1.6. Viscosity

Functions for the confidence interval of 95%, liquid state:

(A17) $$\begin{array}{c} \begin{array}{c} exp(-5.353284065+2026.013242/T\pm 2.262157163\cdot (0.133129577+\\ -83.58237184/T+13127.0558/T^{2}{)}^{1/2}) \end{array} \end{array}$$

Functions for the confidence interval of 99%, liquid state:

(A18) $$\begin{array}{c} \begin{array}{c} exp(-5.353284065+2026.013242/T\pm 3.249835542\cdot (0.133129577+\\ -83.58237184/T+13127.0558/T^{2}{)}^{1/2}) \end{array} \end{array}$$
